# Microfacies impacts on reservoir heterogeneity of early Cretaceous Yamama carbonate reservoir in South Iraq

**DOI:** 10.1038/s41598-024-74640-w

**Published:** 2024-10-15

**Authors:** Abbas Mohammed, Felicitász Velledits

**Affiliations:** 1 Institute of Exploration Geosciences, University of Miskolc, Miskolc, 3515 Hungary; 2 Fields Division, Geology Department, Missan Oil Company, Misan, 62001 Iraq

**Keywords:** Yamama Formation, Reservoir quality, Microfacies, Lithocoduim-Bacinella, Geology, Sedimentology

## Abstract

Detailed well-log interpretations, including gamma-ray, density, neutron, and resistivity, alongside petrographic analysis of 100 samples over 170 m of drill cores, have revealed factors influencing reservoir heterogeneity in the Yamama Formation, Ah’Dimah Oilfield, southern Iraq. The formation comprises four reservoir units (YA-YD) separated by four non-reservoir units (BA-BD). The reservoir units are subdivided into subunits. YB2, YB3, and YC demonstrate the best reservoir quality, while YD2 is water-bearing. Seven microfacies were identified within both reservoir and non-reservoir units, deposited in a shallow carbonate ramp. These include bioclastic wackestone, Lithocodium-Bacinella float/boundstone, peloidal cortoid intraclast grainstone, reefal bioclastic rudstone, bioclastic foraminiferal wacke/packstone, miliolidal pack/grainstone, and spiculitic foraminiferal wackestone. Despite the deep burial depth of the formation (> 4000 m), it maintained good porosity values in most intervals, reaching up to 20%. Early isopachous cement protected porosity and dissolution enhanced porosity, while cementation, compaction, and pyritization reduced it. The reservoir units correlate with depositional environments, being deposited in the shoal area, while non-reservoir units were deposited in lagoon, middle, and outer-ramp settings. The Lithocodium-Bacinella float/boundstone and reefal bioclastic rudstone facies, forming reefal patches and build-ups within the shoal, dominated in YB2 and YC. Targeting these patches northeast of Ah’Dimah Oilfield is promising for field development.

**Supplementary Information:**

The online version contains supplementary material available at 10.1038/s41598-024-74640-w.

Reservoir quality (porosity and permeability) in carbonate rocks is a critical parameter of hydrocarbon exploration and production, where the complexity of microfacies within the carbonate formations exerts a high influence^1,2^. The variations in microfacies, including grain types, grains arrangement, and pore structures/types, play a pivotal role in controlling reservoir quality in carbonate rocks^3^. Additionally, diagenetic processes, such as cementation, dolomitization, and dissolution, can either enhance or destroy the reservoir quality. These diverse processes can lead to the development of several porosity types and permeability, further influencing the overall reservoir quality. Characterizing these variations and understanding the relationship between microfacies and reservoir quality are essential for effective reservoir management, hydrocarbon exploration, and recovery strategies in carbonate rock formations.

Several studies have emphasized the significant impacts of microfacies on reservoir quality in carbonate formations^1,4,5^. The microfacies components, such as the presence of grain-supported versus mud-supported microfacies substantially affect porosity and permeability^3^. These variations are attributed to the type and arrangement of grains, cement, and the prevalence of pore types, including interparticle, moldic, and vuggy porosities. Additionally, micrite envelopes (cortoids) prevent the granules from dissolution thus influencing the reservoir quality^5^. The dissolution may lead to the development of channel porosity by connecting the vuggy and/or moldic porosity, and thus, enhancing permeability. Lucia^4^ has investigated the relationship between microfacies and petrophysical properties. He stated that the composition and texture of microfacies, such as the presence of well-sorted bioclasts, peloids, or intraclasts, can significantly affect porosity and permeability. The abundance of specific components, such as foraminifera, algae, or echinoderms, can further contribute to variations in reservoir quality within carbonate rocks. Moreover, diagenetic alterations, including dissolution, cementation, dolomitization, and pyritization, can either enhance or reduce reservoir quality by occluding or enhancing pore spaces. Mechanical and chemical compaction (pressure solution/stylolite) play a crucial role in the early and late diagenetic history of microfacies. Stylolite’s interaction with cements can influence reservoir quality significantly^1^.

This study aims to provide a comprehensive evaluation of the Early Cretaceous, Yamama Formation’s reservoir heterogeneity in south of Iraq, emphasizing the pivotal role played by microfacies types and the depositional environment. Understanding these factors is of paramount importance for optimizing hydrocarbon exploration and production strategies.

The reservoir quality of the Yamama Formation has been affected by different diagenetic processes including micritization, cementation, recrystallization, silicification, and stylolite formation^6–8^. Therefore, it is challenging to identify the best reservoir units that extend in the Yamama Formation due to the microfacies’ variation and diagenetic processes.

## Tectonic and geologic setting

**Fig. 1:**
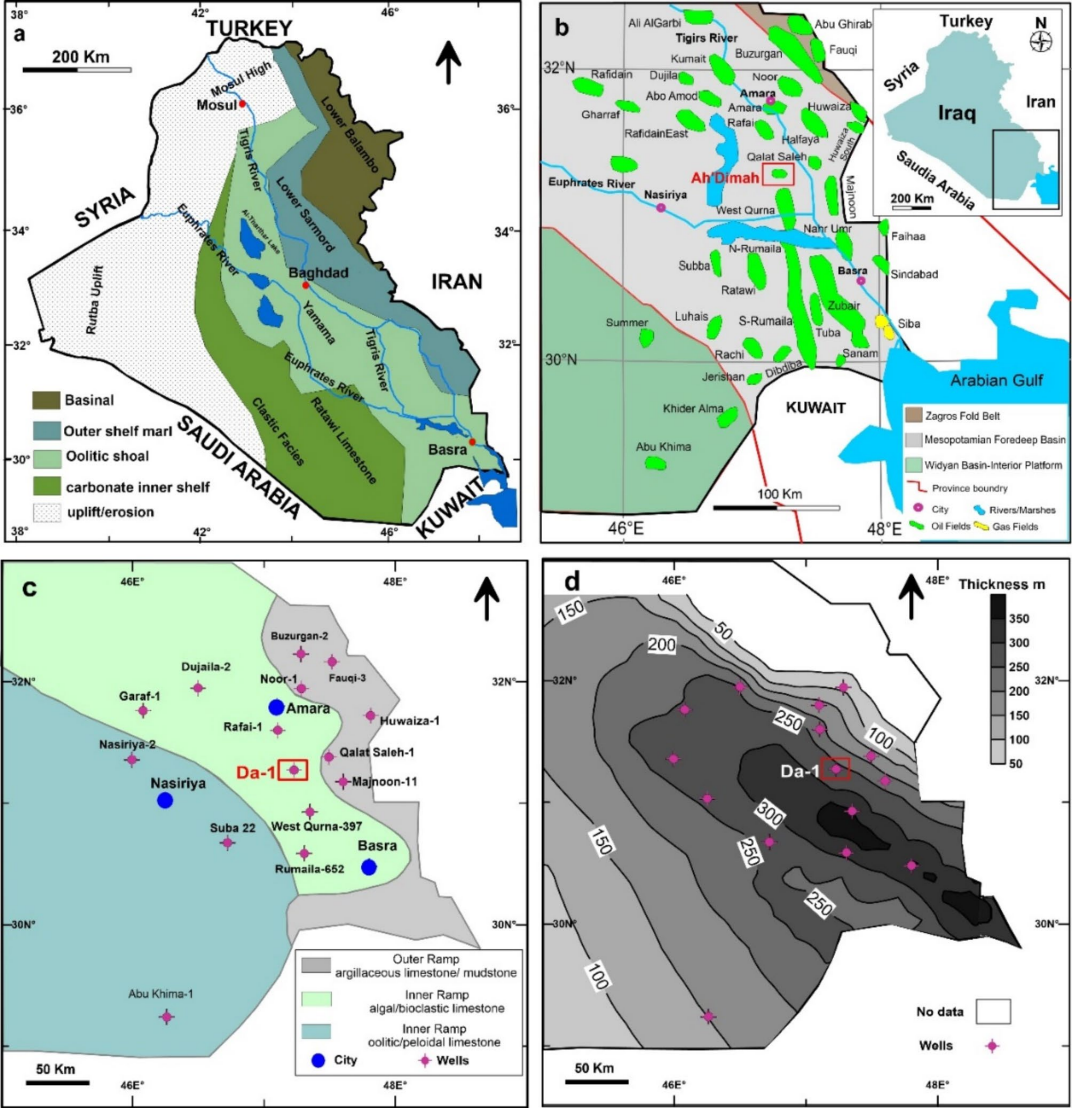
(**a**) Paleographic map of Valanginian age (adapted and regenerated from^13^). (**b**) A location map shows gas/oil fields in south of Iraq. Ah’Dimah Oilfield (Da) of this study is highlighted by red square. (**c**) Facies distribution map of the Yamama Formation in south of Iraq (adapted and regenerated from^16^) with be adapted from ^16,17^. (**d**) Isopach map of the Yamama Formation in the south of Iraq. The maps created using Surfer16.3.408 https://www.goldensoftware.com/.

Iraq is divided into Zagros Thrust Zone, Zagros Flod Belt, Mesopotamian Foredeep Basin (MFB), Widyan Basin-Interior Platform, Rutba Uplift, Khlesiha Uplift, Anah Graben, and Wadi-Surhan Basin^9^. Most petroleum accumulations of the southern and central parts of Iraq are trapped in the buried anticlines of the MFB (Fig. 1S). Iraq’s MFB holds established oil reserves exceeding 100 billion barrels^10^. The MFB includes, the Zubair subzone (southern part of the basin) which contains diapiric structures, Euphrates subzone (western part of the basin), Tigris subzone (the eastern part of the basin)^11^.

The Late Tithonian to Late Cenomanian periods witnessed a southward movement followed by a northward shift of the Arabian Plate during the Albian. This led to the opening of the Southern Neo-Tethys Ocean (SNTO) along the northern and eastern margins of the plate in the Late Tithonian^12,13^. Consequently, a significant regional unconformity occurred during the Tithonian (149 Ma). This unconformity marks the boundary between the mega sequences AP7 and AP8^12^. This likely resulted in a gradual shift in climate, transitioning from the dry conditions of the Late Jurassic to the tropical and humid climate of the Late Cretaceous. In the south of Iraq, the deposition of the basinal evaporites represented by the Gotnia Formation abruptly ceased, allowing the deposition of fine-grained and mud-supported sediments of the Sulaiy Formation.

The AP8 mega sequence includes 6 main cycles. The lower cycle (Late Tithonian – Middle Valanginian) contains the Sulaiy, Yamama and Ratawi in south of Iraq, which is equivalent to the Makhul and Zangura Formations in the central Iraq, Garagu, Sarmord, Karimia and Chia Gara in the Northern Iraq (subsurface), and Garagu, Sarmord, Balambo and Chia Gara in the Northern Iraq (outcrops) (Fig. 1a). This cycle includes three maximum flooding surfaces. These are K30, K20 and K10^12^. The Yamama Formation is also equivalent to the Garau and Lower Fahliyan formations of the Lurestan, Interior Fars, and Persian Gulf of Iran^14,15^.

### Area of study and the Yamama Formation

This study was conducted on the Yamama Formation in Ah’Dimah Oilfield. Ah’Dimah Oilfield is located in the south of Iraq and lies on the MFB, Tigris subzone (Fig. 1b). The Ah’Dimah Oilfield is located around 70 km south of Amara City and some about 10 Km north of West Qurna Oilfield. In 2012, one exploratory well was drilled in Ah’Dimah Oilfield (Da-1) targeting the Cretaceous reservoirs and penetrating the Yamama Formation. The top of the Sulaiy Formation was not reached as reported by the final geological reports. The field is surrounded by giant oilfields such as West Qurna to the south, Halfaya to the northeast, and Majnoon to the east. These oilfield are buried beneath the MFB by the Quaternary cover^18^. Gentle folds trending N-S and NW-SE form these oilfields. However, the seismic survey revealed that the Ah’Dimah Oilfield is an anticline with unclear axes. The northeastern flank of the anticline is more steeply dipping than the opposing limb. The top of the formations in Ah’Dimah Oilfield are higher than their equivalent in Rifaee and lower than in West Qurna Oilfield.

**Fig. 2:**
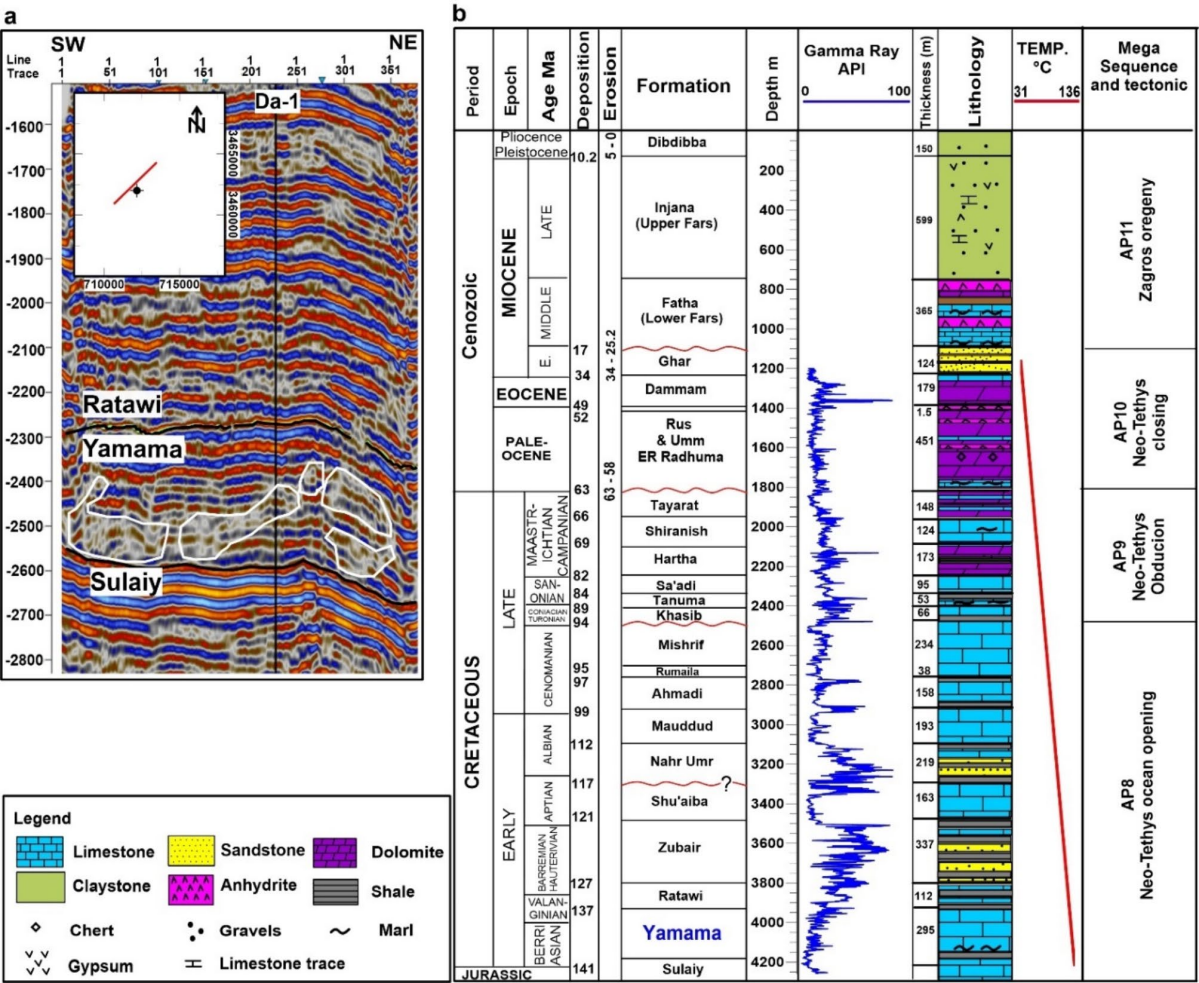
(**a**) Seismic section passing through Ah’Dimah-1 well (Da-1), the Yamama Formation of this study shows build ups within the formation as highlighted with polygons. (**b**) Detailed stratigraphic column of Da-1. The Yamama Formation of this study highlighted with blue.

The Yamama carbonates extend over the Arabian Peninsula^19^. They were deposited on a regional broad low-angle carbonate ramp that developed and prograded basinward (NE) in the Ah’Dimah and West Qurna area during the Early Cretaceous. Yamama deposition probably occurred in a semi-arid environment as tectonic plate movements resulted in the gradual regional climate transition from arid during the Late Jurassic to tropical during the Middle and Late Cretaceous^20^.

The Yamama Formation contains promising reservoir units in the south of Iraqi’s oilfields. The age of Yamama is of the Lower Cretaceous, Valanginian to Berriasian^19^. In Ah’Dimah Oilfield, the Yamama Formation conformably overlies the fine-grained Sulaiy limestone Formation and underlies the Ratawi Formation (Fig. 2). The Yamama Formation showed good reservoir properties in most parts of southern Iraq region including Nasiriya, West Qurna, Halfaya, and Majnoon oilfields. The formation became dry and a potential source rock in Qalat Saleh and Noor oilfields. The Yamama Formation can be a good source rocks for its reservoir units as well as for the overlying Cretaceous formations in the MFB^21,22^.

Three major lithofacies, including argillaceous limestone mudstone of the outer ramp, algal bioclastic limestone of the inner ramp, and oolitic, peloidal limestone of the inner ramp were noted^16^ (Fig. 1c). Similarly, lagoonal, patches reef, shoal and open marine facies were recognized in the Yamama Formation representing a slightly sloping depositional setting as a homoclinal ramp in the Persian Gulf of Iran^7^.

Many different microfacies types were reported by several authors in the Yamama Formation. Mudstone, algal-bearing wackestone-packstone, large foraminifera wackestone-packstone, oolitic and peloidal packstone-grainstone, lithoclastic wackestone-packstone and stromatoporoid sponge-coral boundstone. These microfacies contained large benthic foraminifera including *Everticyclammina sp.p*, *Orbitolina spp*., *Trocholina spp*., *Spirocyclina spp*., *Nautiloculina oolithica*, and *Pseduochrysalidina spp*^7,19,23–25^.

The thickness of the Yamama Formation in the southern part of Iraq varies considerably, ranging from 80 m to the east of Amara City in Noor Oilfield, to over 350 m near West Qurna Oilfield (Fig. 1d). This variability is a result of complex geological processes and localized depositional conditions, which have influenced sediment accumulation over time.

## Materials and methods

A total of 100 samples were collected from approximately 170 m continuous drill cores that were taken from the upper half part of the Yamama Formation in well DA-1 for this study. No cores were taken for the lower half part of the formation. Borehole data, such as final geological reports, well logs (gamma ray, sonic, density, neutron, and resistivity logs), and a seismic section were also collected. Core samples were sent to the laboratory for thin sections preparations. Most of the samples were oil impregnated, therefore, the samples were cleaned with light benzene. Well logs data were processed and interpreted, to reveal the petrophysical properties, and to divide the formation into reservoir and non-reservoir units. Petrophysical properties were calculated empirically from well logs, such as: total porosity, effective porosity, sonic derived porosity, volume of shale, water saturation using mathematical equations. Primary and secondary porosities were determined by the analysis of well logs. The total porosity, which encompasses both primary and secondary porosities, was calculated using density-neutron logs^26^.

The total porosity ($\:PHIT$) is derived from density-neutron logs and is given by the formula:

1 $$\:PHIT=\:\frac{PHID+PHIN}{2}$$

$\:PHID$ represents porosity-derived density, and $\:PHIN$ is porosity obtained directly from neutron porosity logs. The calculation of porosity-derived density ($\:PHID$) is presented below^26^.

2 $$PHID=\frac{{\uprho}\text{m}\text{a}-{\uprho}\text{b}}{{\uprho}\text{m}\text{a}-{\uprho}\text{f}\text{l}}$$

ρma denotes the density of the matrix, the Yamama Formation predominantly composed of limestone, is assumed to be 2.71 g/cm^3^ (the density of calcite). ρb is the density from the density log, and ρfl represents fluid density, which is assumed to be the density of oil (0.9 g/cm^3^).

The effective porosity calculated from the total porosity ($\:PHIT$) as follows^27^.

3 $$\:PHIE=PHIT*(1-Vsh)$$

Shale volume ($\:Vsh$) is determined using Larionov’s equation:

4 $${\text{Vsh}} = 0.33{\text{*}}(22^{{2*{\text{GRI}}}} - 1)$$

$\:GRI$ is the gamma-ray index obtained from the gamma-ray log using the following equation. 

5 $$\:GRI=\:\frac{GRlog-GRlow}{GRhigh-GRlow}$$

$\:GRlog$ represents the natural gamma-ray log reading, $\:GRlow$ is the lowest gamma-ray reading from the gamma-ray log, and $\:GRhigh$ is the highest reading.

Porosity-derived sonic data is calculated using Wyllie’s equation^28^.

6 $$\:PHIS=\:\frac{T\:-\:tma}{Tfl-\:Tma}$$

$\:T$ represents acoustic transit time from the sonic log, $\:Tma$ is the acoustic transit time for the matrix (assumed to be calcite at 47 us/ft), and $\:T$fl. is the acoustic transit time for the fluid (assumed to be 188 us/ft).

Subsequently, the porosity-derived sonic data is empirically corrected for shale and oil effects^27^.

7 $$\:PHIScor.=PHIS*\left(1-Vsh\right)*0.9$$

Water saturation was calculated from resistivity logs based on Archie’s equation^29^ as follows:

8 $$\:Sw={\left(\frac{a*Rw}{{PHIE}^{m}*Rt}\right)}^{\frac{1}{n}}$$

$\:PHIE$ is the effective porosity (Eq. 1), $\:Rw$ is the formation water resistivity, $\:Rt$ is the bulk resistivity obtained from the true resistivity log (deep resistivity), $\:a$, $\:m$ and $\:n$ are the tortuosity factor (assumed 1.3), cementation factor (assumed 1.9), and the saturation exponent (assumed 2), respectively following^30^.

Sixty-five plug samples porosity and permeability measurements were provided by the Oil Exploration Company, Ministry of Oil, Iraq. The formation was subdivided into reservoir and non-reservoir units based on well logs signals and petrophysical properties such as porosity, volume of shale and water saturation. The reservoir and non-reservoir discrimination was mainly based on the porosity, following the classification of Nabawy and Al-Azazi^31^ and Nabawy et al.^32^ with consideration of the volume of shale and water saturation. Intervals with a porosity ≤ 10%, volume of shale ≥ 20%, and water saturation ≥ 30% are considered reservoirs, and vice versa for the non-reservoir intervals. Macro-analysis was carried on the core samples to reveal the lithology, fossils content, visible pores, oil shows, and other sedimentological structures. The Samples were stained with blue resin, examined and classified based on characteristics such as mineralogy and texture (according to Dunham’s classification^33^), pore system according to (Choquette and Pray^34^, and diagenetic features. The standard microfacies classification (RMFs) of Flügel^5^ were followed to classify the microfacies in this study and revealed the depositional environments.

## Results

### The Yamama Formation zonation

The results show that the Yamama Formation in Ah’Dimah Oilfield is heterogenous and consists of limestone, argillaceous limestone, and fossiliferous limestone (Fig. 2S). The intervals with porosity > 10% and with low volume of shale values are considered porous limestone, porosity of about 10 − 5% with relatively a higher amount of volume of shale considered as argillaceous limestone, and the intervals with porosity > 5%, fossils contents and relatively clean are considered as fossiliferous limestone. The non-reservoir units consist of fossiliferous limestone interbedded with argillaceous limestone. Whereas the reservoir units consist of mainly limestone with some argillaceous limestone.

Yamama unit is a seal for the Yamama Formation. BA, BB, BC, and BD are non-reservoir units that split the YA, YB, YC and YD reservoir units (Fig. 2S). YA shows fair porosity in the upper part with poor overall averaged porosity (Fig. 2S; Table 1). YB was subdivided into three reservoir subunits (YB1, YB2 and YB3) that separated by non-reservoir beds (Table 1). YB2, YB2, YB3 and YC form the best reservoir unit in the Yamama Formation. Non-reservoir layers were also observed within the main reservoir units YB1, YB3, and YC (Fig. 2S). This indicates that the Yamama Formation is highly heterogeneous. YD is subdivided into two subunits, YD1 shows good porosity but with a relatively higher water saturation compared to other reservoir units, while YD2 shows higher porosity and higher water content values, thus YD1 is the transition zone and the border between YD1 and YD2 is highly likely the oil water contact (OWC). Based on the classifications of Nabawy and Al-Azazi^31^ and Nabawy et al.^35^, the porosity of the reservoir units in the Yamama Formation ranges from poor-fair-good and the permeability ranges from poor to fair. While the porosity and permeability in the non-reservoir units are classified as impervious.

**Table 1:** Well logs and core measurements derived petrophysical properties in each reservoir and non-reservoir unit of the Yamama Formation.

Unit	Average						Thickness m	Description
	PHIE %	PHIS %	Core PHI %	Core K mD	Vsh %	Sw %		
Yamama	5.8	4	-	-	27	35	19.66	Non-reservoir
YA	7.8	7.3	6.9	3,2	20	24	20.88	Poor reservoir
BA	3.8	3.7	-	-	25	33	6.25	Non-reservoir
YB1	10	8.1	14,4	3.5	17	22	30.00	Fair reservoir
YB2	15.7	13.3	19	9	10	22	27.28	Good reservoir
YB3	10	8.5	15.5	12	15	30	19.35	Fair-good reservoir
BB	6.6	4.9	-	-	13	27	12.34	Non-reservoir
YC	12	10	17	13	13	27	19.66	Good reservoir
BC	6.2	5.7	-	-	16	55	27.43	Non-reservoir
YD1	11	9.1	-	-	7	43	20.27	Fair Reservoir
YD2	12.3	9.9	-	-	6	66	59.44	Water-bearing
BD	5.4	4.6	-	-	13	78	43.28	Non-reservoir

PHIE = effective porosity, PHIS = sonic porosity, core PHI and core K = core porosity and permeability measurements respectively, Vsh and Sw = volume of shale and water saturation derived from well logs, respectively.

### Microfacies analysis

**Fig. 3:**
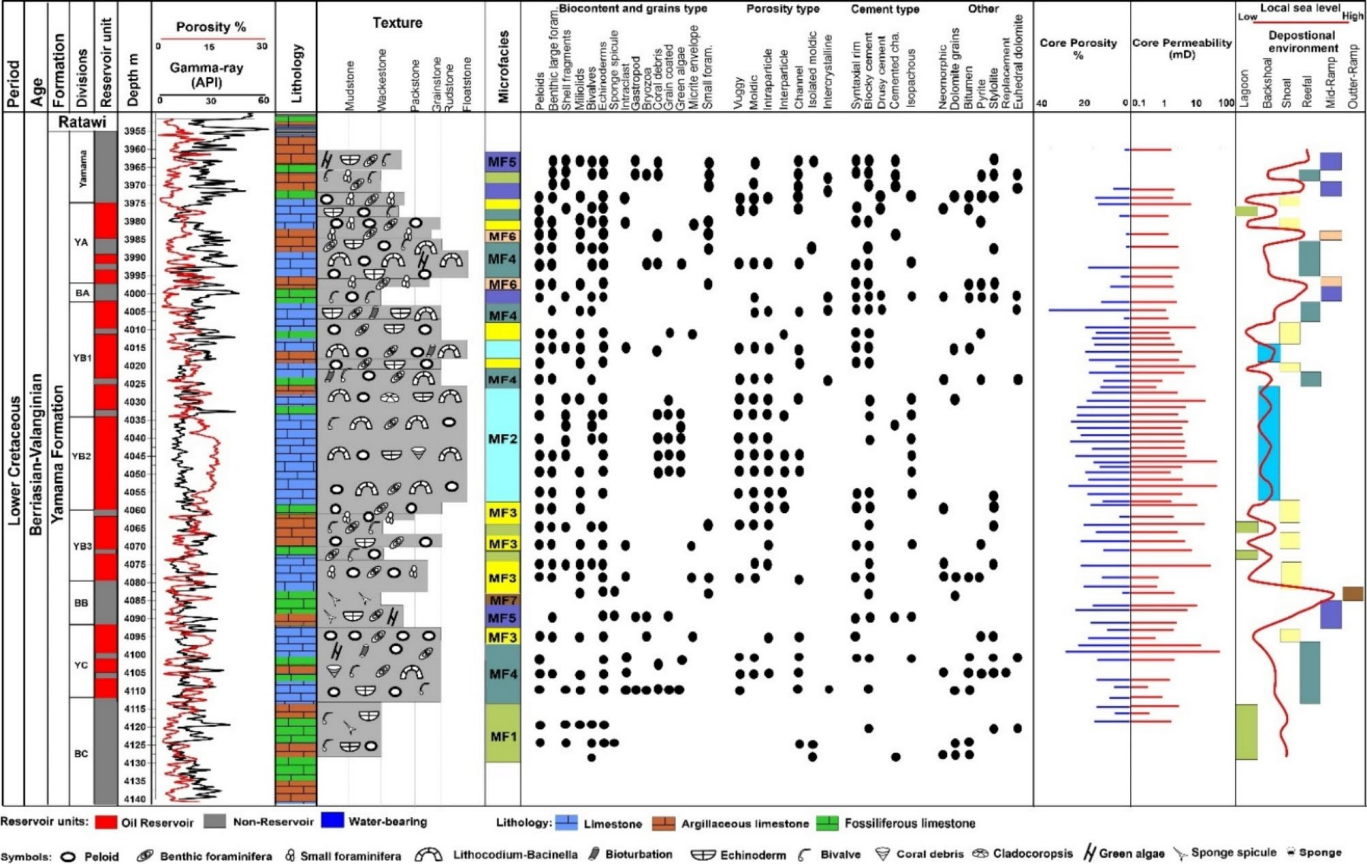
Detailed sedimentological log of the Yamama Formation, showing the vertical microfacies’ variation, microfacies analysis, core porosity-permeability measurements, and the depositional environments of each microfacies.

Examination of the thin sections from the Yamama Formation within the Ah’Dimah Oilfield, conducted under microscopic analysis, has unveiled the presence of vertical heterogeneity in the formation. A total of seven distinct microfacies types have been observed (Fig. 3; Table 2). These microfacies include the bioclastic wackestone (MF1), Lithocodium-Bacinella float/boundstone (MF2), peloidal cortoid intraclast grainstone (MF3), reefal bioclastic rudstone (MF4), bioclastic foraminiferal wacke/packstone (MF5), miliolidal pack/grainstone (MF6), and spiculitic foraminiferal wackestone (MF7). Large foraminiferas, Lithocoduim-Bacinella algae, bivalves, coral, and echinoderms are common. Conversely, smaller foraminiferas, bryozoa, gastropods, and sponge spicules are comparatively less prevalent. Additionally, the Yamama Formation is commonly characterized by the presence of non-skeletal grain types, such as peloids, cortoids, and intraclasts.

**Table 2:** Microfacies’s description, facies associations, percentage, thickness, averaged effective (PHIE) and sonic log porosity (PHIS), volume of shale (vsh), porosity (PHI) and permeability (K) measurements of the plug samples.

Microfacies	Composition	Energy level	Facies association	Thickness m	Average			
					PHIE %	Vsh %	Core PHI %	Core K mD
MF1: Bioclastic wackestone	Thick shells and bivalve fragments, intermittent sponge spicules, minor peloids and echinoderms	Low-moderate	Lagoon	25.7	7	14	12.7	2.2
MF2: Lithocodium-Bacinella float/boundstone	Lithocodium-Bacinella, shell fragments, echinoderms, and coral debris. Occasionally occurred peloids and intraclasts	Moderate- high	Backshoal	39	13	13	16.7	7.2
MF3: Peloidal cortoid intraclast grainstone	Fine-coarse, rounded-subrounded, peloids and cortoids are dominant, intraclasts, large foraminifera, echinoderms and brachiopods	High energy influenced by wave or/and tide	Shoal barrier	33.4	10	14.8	14.6	10.8
MF4: Reefal bioclastic rudstone	Lithocodium-Bacinella, coral, Caldocoropsis, large foraminifera, gastropods, echinoderms, bryozoa, also peloids and intraclasts occasionally occurred	High energy, influenced by tide	Shoal reefal patches	37.2	10	15	15	9.1
MF5: Bioclastic foraminiferal wacke/packstone	Bioclasts, large and small foraminiferas, echinoderms, sponge spicules	Low-moderate	Middle ramp	25	5.5	21	3.8	1.7
MF6: Miliolidal pack/grainstone	Well sorted, fine peloids and cortoids with abundance of the small size foraminiferas	Low	Middle ramp	3.6	4.6	21	-	-
MF7: Spiculitic foraminiferal wackestone	Sponge spicules and small foraminiferas	Low	Outer ramp	4.1	5.8	8	-	-

## Lagoonal facies association

### Bioclastic wackestone MF1

**Fig. 4:**
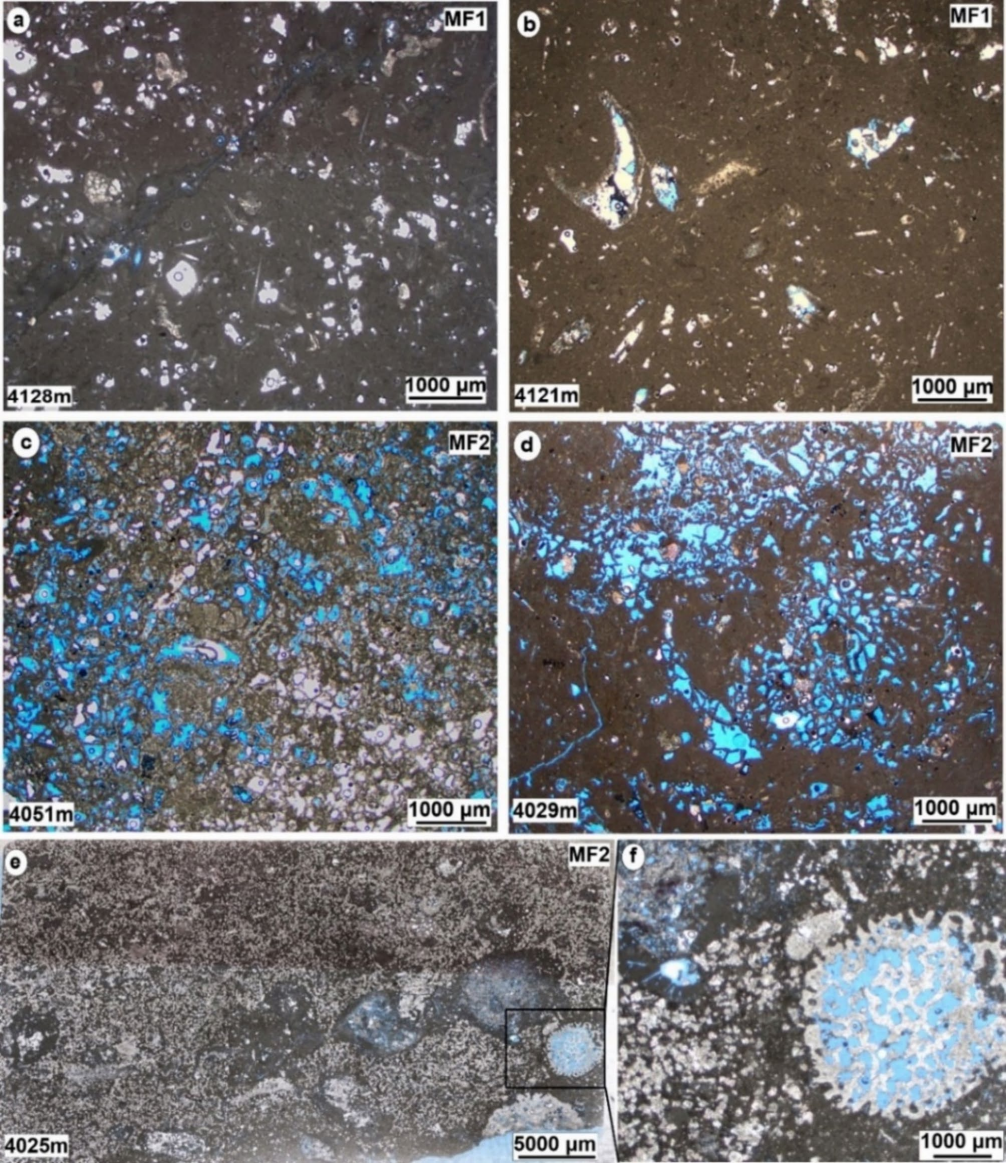
Micrographic photos of the Yamama Formation shows lagoonal and backshoal microfacies. (**a** and **b**) Bioclastic wackestone (MF1). (**c** and **d**) Lithocodium-Bacinella float/boundstone (MF2). (**e**) A scanned micrographic photo shows dolomitization within MF2. (**f**) Zoomed of the highlighted rectangle in **e** shows cladocoropsis within MF2. All photos are under plane polarized light (PPL).

**Fig. 5:**
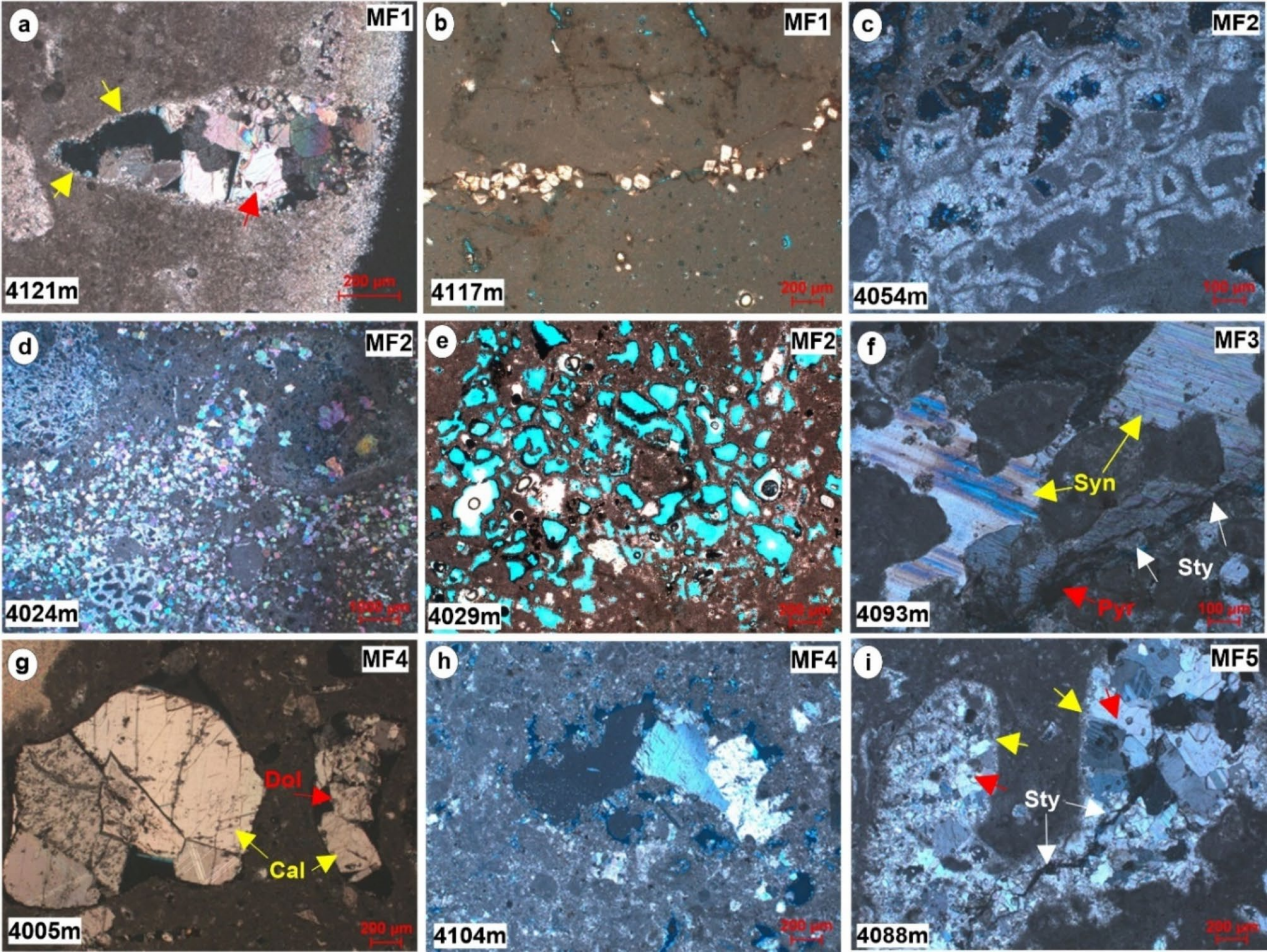
Micrographic photos show some diagenetic features in the Yamama Formation. (**a**) Partially cemented moldic pore with two cement generations, fine crystalline cement (yellow arrows) along the mold’s edge followed by blocky calcite cement (red arrows). (**b**) Dolomite rhombs along stylolite. (**c**) Isopachous cement surrounding Lithocodium-Bacinella. (**d**) Dolomitization. (**e**) Dissolution. (**f**) Syntaxial cement (yellow arrows), pyrite (red arrow) and stylolite (white arrows), (**g**) Calcite and dolomite filled moldic pores. (**h**) Partially cemented moldic pore. (**i**) Completely calcite cemented moldic pores with two cement generations, fine crystalline cement (yellow arrows) followed by blocky calcite cement (red arrows), it also shows stylolite crosses the cement (white arrow). Cal = calcite cement, Dol = dolomite, Sty = stylolite. All photos are under crossed polarized light (XPL), except **b** and **e** (under plane polarized light PPL).

*Description*. This particular microfacies is observed at the lowermost part of the cored interval within the Yamama Formation. It is characterized by the presence of an abundance of thick shells and bivalve fragments, as well as bioclasts (Fig. 4a and b). This facies comprises up to 85% of the mud-matrix. Furthermore, a certain proportion of argillaceous limestone is notable within this facies, with intermittent occurrences of sponge spicules in some intervals. Minor quantities of peloids, along with relatively large foraminifera including miliolids as well as echinoderms, are also presented, while smaller foraminifera are absent. Bioturbation is common in this facies type. The extent of this microfacies is approximately 25.75 m, constituting 15% of the entire cored intervals. The porosity varies from 1.7 to 16.5% with an average of about 7%. Predominant porosity types, including moldic, intraparticle, and channel were found within this type of facies. Most moldic pores are observed completely or partially cemented with two cement generation (Fig. 5a). It is worth noting that the channels exhibit blocky cementation, with the prevalence of such blocky cement being a distinctive feature. This microfacies is predominantly found in the non-reservoir units of the Yamama Formation situated at the base of the cored interval and show poor reservoir quality. Dolomite grains are common along the stylolite or fractures (Fig. 5b). These stylolites arise from pressure solution processes, as a consequence of chemical compaction subsequent to the mechanical compaction stage during the late diagenetic processes^1^.

### Backshoal facies association

#### Lithocodium-Bacinella float/boundstone MF2

**Fig. 6:**
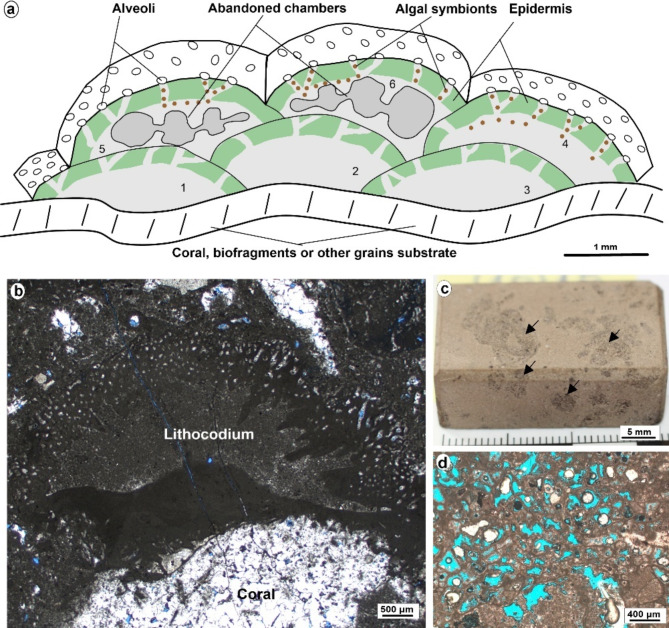
(**a**) a schematic diagram of the Lithocodium-Bacinella, showing 6 chambers (modified after Schmid and Leinfelder 1996). (**b**) A micrographic photo showing typical cross-section view of Lithocodium-Bacinella that encrusting on coral. (**c**) A core photo showing the Lithocodium-Bacinella facies. (**d**) A micrographic photo shows a plan view of the vuggy porosity within Lithocodium-Bacinella facies.

*Description*. The predominant skeletal grains within this facies are the Lithocodium aggregatum- Bacinella irregularis, which are observed floating on a mud-dominated matrix (Figs. 4c–f and 6). Additionally, this facies includes common skeletal components such as large benthic foraminiferas, including Miliolids, *Pseudochrysalidina*, and *Pseudocyclammina littus*, along with shell fragments, echinoderms, and coral debris. Peloids and intraclasts are also noted within this facies. This significant facies encompassing 23.2% of the overall cored intervals within the Yamama Formation. It boasts a considerable thickness of approximately 39 m, making it the most extensive among the various facies. Remarkably, this facies shows good reservoir quality within the Yamama Formation. Intervals with Lithocodium-Bacinella exhibit higher porosity values and a lower shale volume, accompanied by varying permeability values when compared to other facies (Fig. 3; Table 2). The porosity in this facies varies from 2.5 to 19.5% with an average of 13%. Considerable dissolution forming vuggy porosity is observed within the Lithocodium-Bacinella bodies (Fig. 5e). The isopachous cement and the dissolution are common in this facies (Fig. 5c). Dolomitization is also noted within MF2 (Fig. 5d).

### Shoal facies association

#### Peloidal cortoid intraclast grainstone (MF3)

**Fig. 7:**
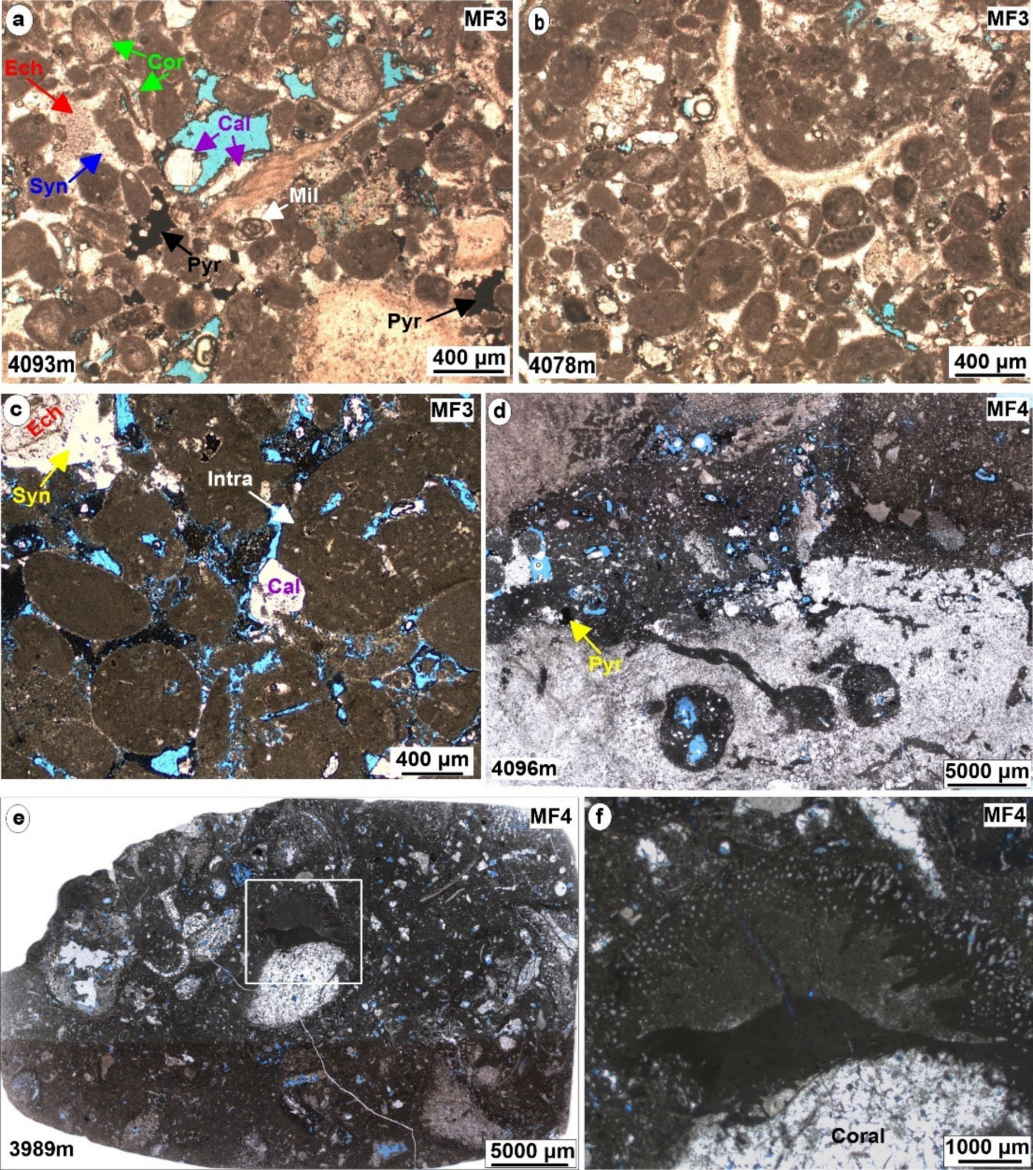
Micrographic photos of the shoal and reefal patches microfacies. (**a**–**c**) Peloidal cortoid intraclast grainstone (MF3). (**d**–**f**) Reefal bioclastic rudstone (MF4). (**f**) A cross-section view shows Lithocodium-Bacinella encrusting on coral fragment that replaced by calcite cement within MF4. Ech = echinoderm, Cal = calcite cement, Cor = cortoid, Syn = syntaxial cement, Mil = miliolid, intra = intraclast, Pyr = pyrite. All photos are under plane polarized light (PPL).

*Description*. Predominantly, this facies is composed of peloids and micrite envelopes, also known as cortoids (Fig. 7a and b). The peloids exhibit a variable grain size range, spanning from fine to coarse grains, typically falling within the 0.2 to 0.5 mm range. These grains generally exhibit subrounded – rounded shape, poor sorting in the samples. This facies includes other components such as large benthic foraminiferas, specifically Miliolids, *Pseudochrysalidina*, *Praechrysalidina*, and *Pseudocyclammina littus*, along with the presence of echinoderms and brachiopods. Additionally, grain aggregate formed by fragments of Lithocodium-Bacinella have also been identified in certain intervals of this facies. This particular facies accounts for approximately 20% of the total cored intervals within the Yamama Formation of Ah’Dimah Oilfield, encompassing a thickness of approximately 33.4 m.

This facies demonstrates good reservoir quality and is exclusively located within the reservoir units of the Yamama Formation (Fig. 3; Table 2). The porosity in this facies varies from 2% to 17.5 with an average porosity of about 10%. The interparticle porosity is dominant, while, intraparticle, vuggy, and channel porosity are less frequently encountered. Various cement types, including blocky, drusy, syntaxial rims, and pyrite nodules are found occupying pore spaces, specifically within vugs and interparticle pores of this microfacies (Figs. 5f and 7a and c). Additionally, certain micrite envelopes exhibit complete blocky and drusy cementation. The presence of stylolites within this facies, cutting through syntaxial cements, indicating both mechanical and chemical compaction. Dolomite rhomb have also been noted in association with the formation of stylolites, and occluded pore spaces in some cases.

### Shoal reefal patches/foreshoal facies association

#### Reefal bioclastic rudstone MF4

*Description*. This particular microfacies extends over a vertical interval of 37.2 m, representing approximately 20% of the entire cored intervals within the Yamama Formation. The predominant components of this microfacies include Lithocodium-Bacinella, coral, sponge and cladocoropsis. Large benthic foraminifera, such as Miliolids, *Pseudochrysalidina*, *Pseudocyclammina littus*, *Trocholina sp*, *Lenticulina sp* in addition to shell fragments, bivalves, gastropods, echinoderms, and bryozoa are common (Fig. 7d-f). This facies exhibits considerable amounts of peloids and intraclasts comprising the non-skeletal grains within this microfacies. Notably, bioturbation and the infilling burrows have been observed within this microfacies.

This microfacies exhibits diagenetic processes involving both euhedral and anhedral dolomite, thereby engendering intercrystalline porosity that is commonly occluded by calcite cement (Fig. 5g and h). Pyritization has occurred within the skeletal grains, as well as within the matrix and cement, contributing to the complex diagenetic history of this microfacies (Fig. 7d). The pervasive cementation, manifesting as isopachous, blocky and syntaxial cements, effectively occludes some pore spaces. Nevertheless, this microfacies presents a good reservoir quality and is predominantly encountered within the reservoir units of the Yamama Formation. The intraparticle and vuggy porosity are dominant. The porosity varies from 1.6 to 18% with an average porosity of about 10%.

### Middle ramp facies association

This facies association includes two facies, Bioclastic foraminiferal wacke/packstone (MF5) and Miliolidal pack/grainstone (MF6).

### Bioclastic foraminiferal wacke/packstone MF5

**Fig. 8:**
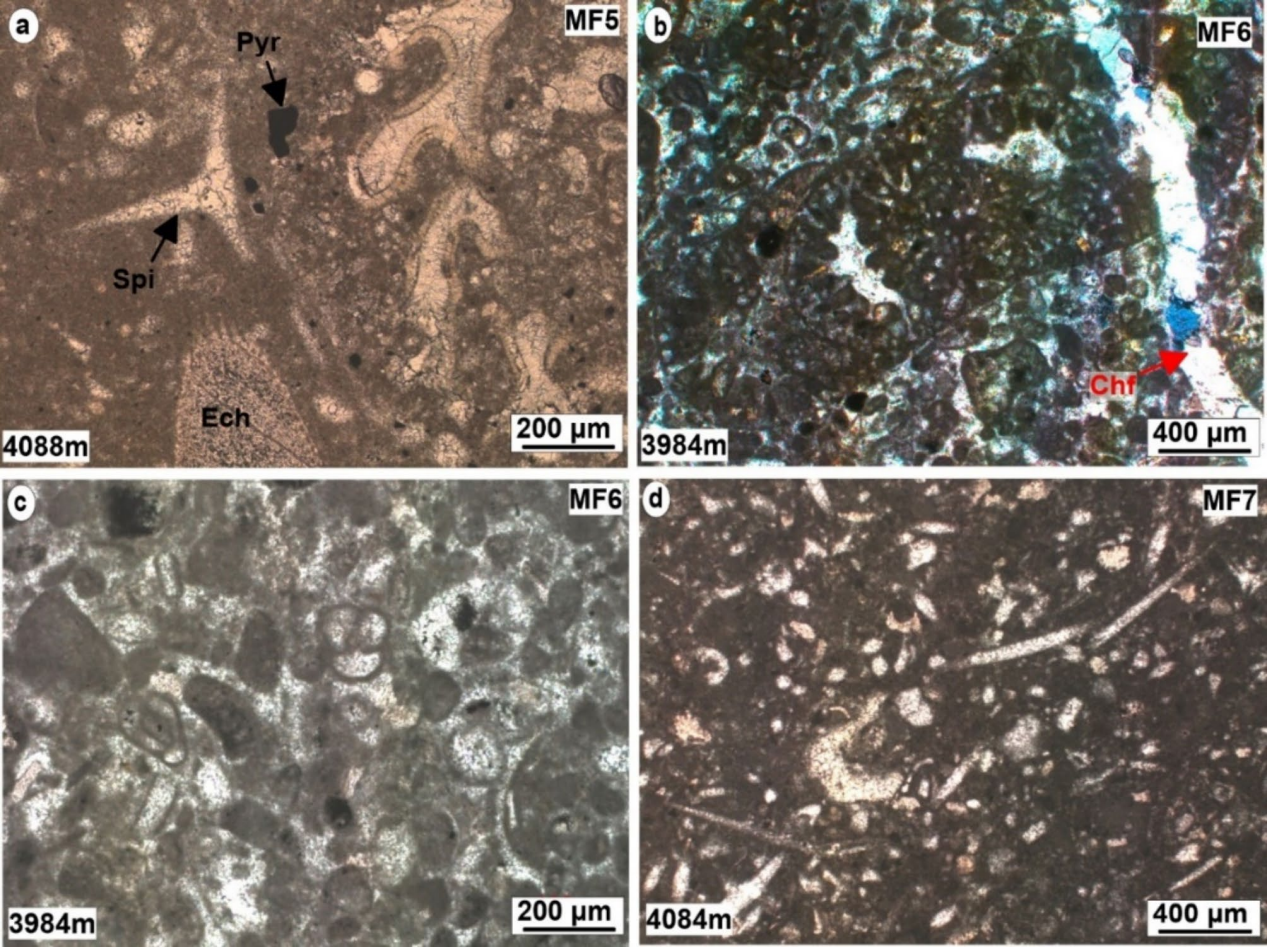
Micrographic photos of the middle and outer ramp microfacies of the Yamama Formation. (**a**) Bioclastic foraminiferal wacke/packstone. (**b**) shows *Pseudocyclammina littus* within Miliolidal packstone/grainstone. (**c**) Shows small foraminiferas with Miliolidal packstone/grainstone. (**d**) shows spiculitic foraminiferal wackestone. (**a**), (**c**), and (**d**) photos are under plane polarized light (PPL), (**b**) is under cross polarized light (XPL). Pyr = pyrite, Spi = sponge spicules, Ech = echinoderm, Chf = channel filled with cement.

*Description*. This particular facies has a wackestone texture and also showed packstone texture in some cases. It is comprised of bioclasts fragments, large benthic foraminiferas such as Miliolids, *Pseudochrysalidina*, *Pseudocyclammina littus*, *Lenticulina sp*, and echinoderms. This facies exhibit notable amount of small foraminiferas as well as sponge spicules, (Fig. 8a). It constitutes approximately 15% of the total cored intervals, with a vertical extent of 25 m. This facies exhibits poorer reservoir quality, marked by low porosity, low permeability, and a higher shale content when compared to other facies within the Yamama Formation (Table 1). The porosity varies from 0.9 to 11% with an average of about 5.5%. The limestone in this facies is often argillaceous. Notably, moldic, vuggy, and channel porosities have been entirely occluded by blocky, syntaxial, and drusy cement, resulting in the reducing of porosity (Fig. 5i). This facies has undergone dolomitization, stylolitization and pyritization. Certain intervals exhibit substantial dolomitization.

### Miliolidal pack/grainstone MF6

*Description*. This facies shows packstone to grainstone texture. The main constituent is the non-skeletal grain of peloids. The peloids are less than 0.2 mm in size and well sorted (Fig. 8b and c). The small Miliolid foraminiferas are common in this facies. This facies shows similar characters of MF3. However, for the importance of evaluating the microfacies and their reservoir quality, this facies was distinguished. The main distinctive feature between the MF6 and MF3 is the size of skeletal and non-skeletal grain size. This facies consists of fine-grained peloids and cortoids, with the abundance of the small size foraminiferas such as miliolids. This type of facies forms 2.14% (3.6 m) of the entire cored intervals of the Yamama Formation. This facies exhibits poor reservoir quality compared to MF3. The porosity in this facies varies from 2 to 7.8% with an average of 4.6%. Notably, this facies shows a higher volume of shale (Table 1).

### Spiculitic foraminiferal wackestone MF7

*Description*. Sponge spicules and small foraminiferas are the main skeletal grains in this facies which form more than 10% of the examined samples (Fig. 8d). The large foraminifera and non-skeletal grain such as peloids, cortoids and intraclast are absent. Small-sized Miliolids and echinoderms occasionally appeared in this facies. It occurred in one interval with thickness of about 5 m and therefore, forms of about 3% of the cored intervals. Blocky and sparite cement were presented in this type of facies. Dolomite grains were found as scattered crystals within the samples. This type of facies shows poor reservoir characteristics. The porosity varies from 5 to 9% with an average of 5.8%. This microfacies was found within the non-reservoir unit BB (Fig. 3; Table 2).

### Porosity-permeability relationships

**Fig. 9:**
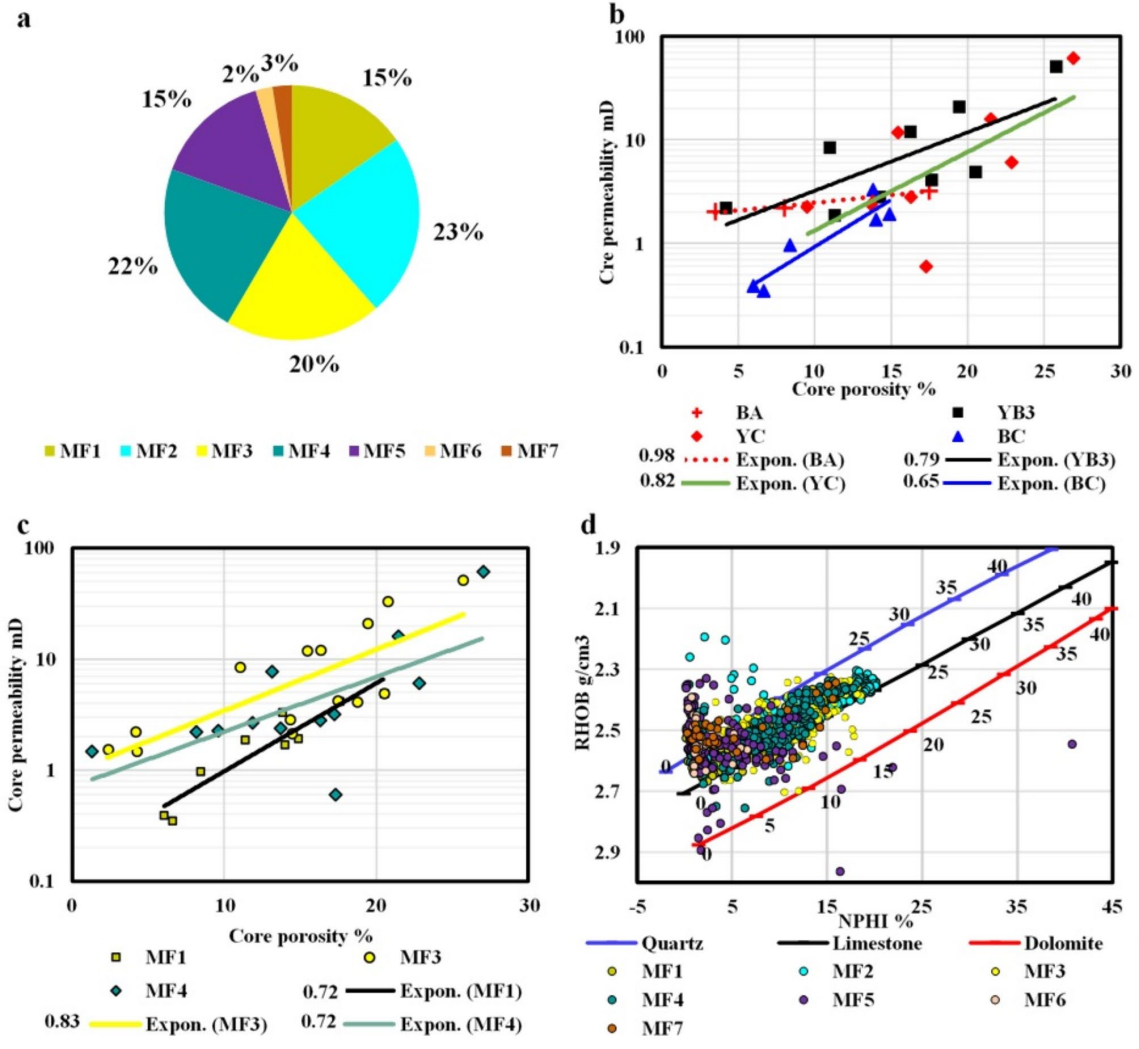
(**a**) Pie chart showing the percentage of each microfacies. (**b**) and (**c**) Core porosity and permeability measurements cross-plots for the various reservoir and non-reservoir units, and for the various microfacies, respectively. The values in the legends represent the coefficient of determination (R^2^) of the exponential best-fit lines, only values with higher than 0.65 are showing. (**c**) Density-neutron cross plot for the various microfacies.

There was no distinct overall relationship observed between porosity and permeability measurements within the Yamama Formation across the various microfacies with depth. The core porosity measurements tended to exhibit somewhat higher values when compared to the porosity values derived from logs (Fig. 2S; Table 2). This since the diagenetic processes affected each microfacies to a different extend. The overall exponential correlation between porosity and permeability measurements yielded a coefficient of determination (R²) of approximately 0.25. However, observable trends and relationships emerged when the formation was divided into the various reservoir and non-reservoir units (Fig. 9b). MF1, MF3, and MF4 show clear exponential relationships between porosity and permeability (Fig. 9c). YA, YB1, and YB2 show limited porosity and permeability relationships. Whereas notable relationships were observed in BA, YB3, YC, and BC (Fig. 9b). For instance, MF2, MF3 and MF4 are the predominant facies in YC and YB3, respectively. These facies primarily consist of peloids and exhibit a substantial presence of interparticle porosity, resulting in elevated porosity and permeability relationships (Fig. 3). In contrast, YA and YB1 exhibit limited porosity and permeability relationships due to their distinct facies compositions. Given that the majority of microfacies identified in this investigation displayed sandy-sized peloids, the well logs exhibited characteristics similar to sand properties when density and neutron logs were cross-plotted. It is noticed that the dissolution played important roles in shaping the overall porosity in MF2, where dissolution can enhance the porosity but not necessarily the permeability. There is no clear porosity and permeability relationship found in YB2, likely because MF2 is dominant in that reservoir unit. However, YB2 shows overall higher porosity and permeability trend when comparing to other facies (Fig. 3). Following the approach of Safa et al.^2^ and Nabawy et al.^31,35^ to divide the formation into hydraulic flow units and reservoir rock types based on porosity-permeability measurements is a valuable method. This approach was not utilized in this study but is recommended for future research to enhance the understanding of the Yamama Formation’s reservoir characteristics.

## Discussion

### Depositional setting of Yamama Formation

**Fig. 10:**
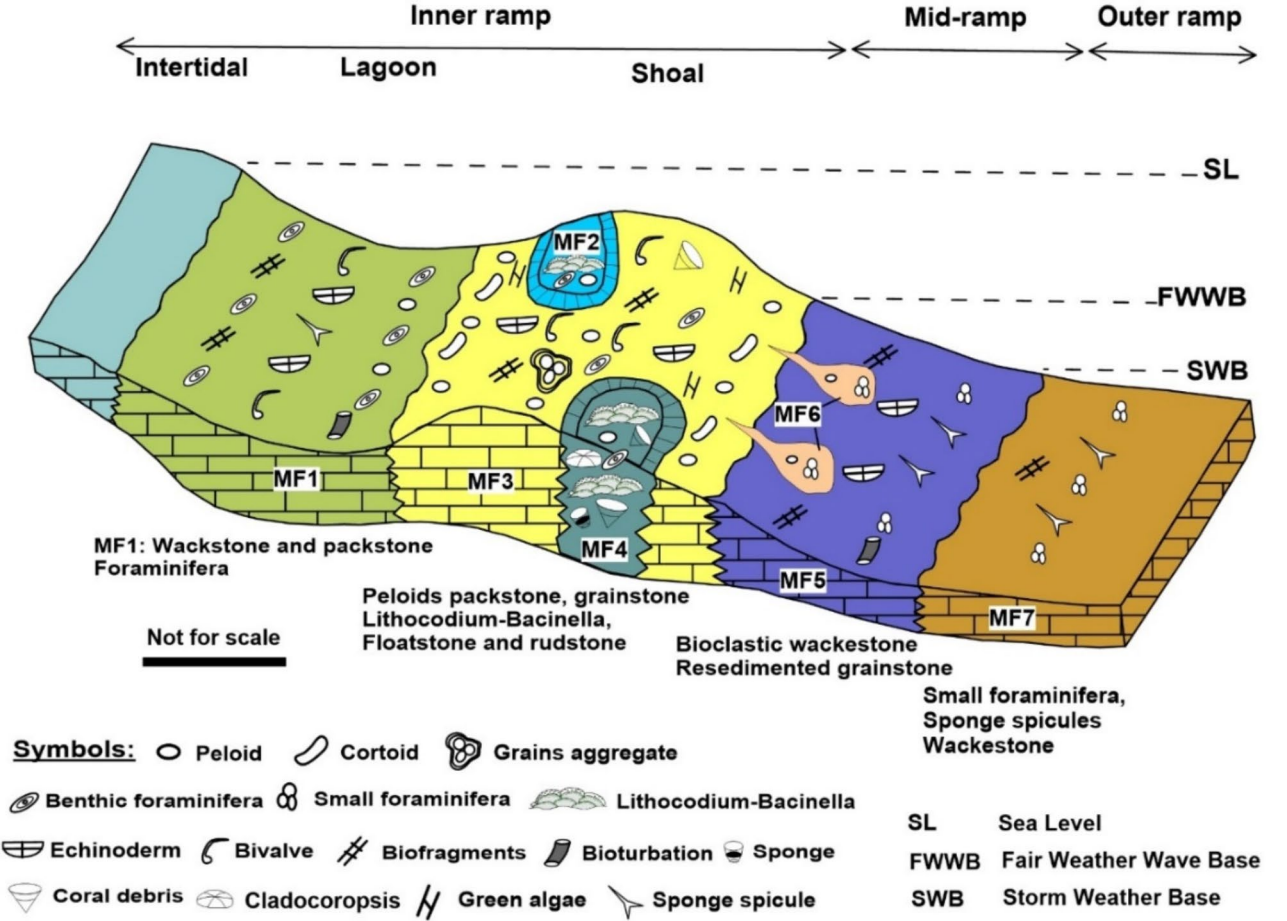
A conceptual depositional environment model for the Yamama Formation showing distribution of the various facies in a carbonate ramp setting.

The depositional environments of the facies within the Yamama Formation are classified into four primary categories: lagoon, shoal, middle ramp, and outer ramp (Fig. 10). Notably, the shoal setting is further delineated into three distinct sub-settings, these are the backshoal, foreshoal, and shoal core or center. However, it is essential to note that no evidence of basinal or intertidal facies were identified in this study.

The vertical variation of facies within the Yamama Formation provides valuable insights into localized sea-level fluctuations. Therefore, sea level changes played significant roles in microfacies’ variation. MF1 is interpreted as having been deposited in a lagoon environment characterized by relatively low to moderate energy conditions. Within MF2, numerous skeletal and non-skeletal grains were observed interacting with the Lithocodium-Bacinella algae in floatstone texture. These constituents include peloids, large foraminiferas, echinoderms, and coral debris, and the prevalence of isopachous cement is notable. These collective observations indicate that this facies was deposited in a moderate to high energy setting and is interpreted to be situated between the lagoon and shoal within the backshoal environment as buildups (Fig. 10).

The preponderance of peloids, cortoids, large foraminifera, as well as brachiopods and other skeletal grains within MF3 points to a high-energy environment and implies deposition in a shoal setting, specifically the shoal core^15^. In contrast, the features observed within MF4 including Lithocodium-Bacinella, sponge, coral, cladocoropsis, as well as the presence of peloids, large foraminifera, echinoderms and bivalves as well as the abundance of isopachous cement suggest a deposition within a high energy environment^5,36^. This facies is situated above the fair-weather wave base (FWWB), influenced by tides, and positioned within the shoal zone, subject to daily high and low tides. This facies represent reefs that form reefal patches within the foreshoal area.

The small foraminifera, the appearance of the sponge spicules, the absence of non-skeletal grains like peloids, in addition to the appearance of the argillaceous limestone in this facies all suggest low energy environment^5,15,37^. This facies is interpreted to have been deposited in middle ramp setting below FWWB and above SWB. The presence of stylolites intersecting the syntaxial cements confirms their deep burial diagenetic origin. MF6 is likely to have been reworked, slopped, and deposited in a lower energy environment compared to MF3 and is presumed to have been deposited within a middle ramp setting. The presence of sponge spicules and small foraminifera in MF7 suggests a deeper depositional environment when compared to other facies, characterized by low energy conditions^37,38^. Consequently, this facies is inferred to have been deposited in the outer ramp setting.

### Microfacies, diagenetic processes and reservoir heterogeneity of the Yamama Formation

The combined core observations and petrographic examination of the thin sections reveal that the Yamama Formation is heterogeneous and consists of various microfacies (seven microfacies). The Yamama Formation’s microfacies experienced various diagenetic processes affecting its reservoir units to different extents (Fig. 5). The formation underwent micritization, dissolution, different types of cementations and generations, pyritization and dolomitization, as well as physical and chemical compaction. These diagenetic processes played pivotal roles in enhancing or destroying the porosity. Despite the deep burial depth of the Yamama Formation in this study (> 4000 m), the formation maintained good porosity values in most of its intervals, reaching up to 20%.

The dissolution significantly contributed to the creation of secondary porosity in the formation, resulting in irregularly shaped vugs, moldic pores, channels, and interparticle porosity. Both fabric-selective and non-fabric-selective dissolution developed the porosity near the phreatic marine zone. In contrast, the different cement generation occluded the pore spaces in some microfacies and thus reduced the porosity.

MF1, MF5 and MF7 showed low porosity and permeability and were found mainly within the non-reservoir units Yamama, BA, BB, BC that separate the reservoir units in the formation. These microfacies showed abundance of different types of cement, pyrite and that occluded the pore spaces as well as compaction (Figs. 5a and i and 8a). The styolite in these types of facies is an indicator to the chemical compaction that influenced the porosity (Fig. 5b and i). Some occurrences of MF1 are encountered within the reservoir units of the Yamama Formation, particularly, in YB3. Therefore, fluctuating the reservoir quality in this reservoir unit (Fig. 3). For the abundance of Lithocodium-Bacinella in MF2 and MF4, and for their importance in shaping the Yamama reservoir, MF2 and MF4 will be further discussed in the next section.

The main type of porosity encountered within the MF3 is the interparticle porosity, found between the peloids and cortoids (Fig. 7a–c). The reservoir quality has been adversely affected by pyritization, cementation, and dolomitization, which have served to occlude pore space in this facies (Figs. 5f and 7a and c). The micritization in the form of micrite envelopes exhibit remarkable resistance to dissolution, acting as protective shields that protect the grains from deterioration. They often play a pivotal role in preserving the structural integrity of original aragonitic skeletal grains, particularly when subjected to freshwater diagenesis^7,32^. Furthermore, these cortoids or micrite envelopes, can serve as indicators of heightened pore fluid pressures, potentially mitigating the effects of lithostatic loading^39^. This microfacies showed abundance of syntaxial cement that formed around echinoderms and occluded the pore spaces (Fig. 5f). MF6 shows pack/grainstone texture, the spar calcite cement found between the fine grains of this facies prior to the mechanical and chemical compaction as well as some fractures were observed filled with cement. These indicate that this facies was affected by late stage of cementation and compaction, hence reduced the porosity (Fig. 8b and c). Fine grained sediments can lose porosity due to compaction more than in the coarse grained sediments^1^.

The density-neutron cross-plot shows that most data points fall on limestone (Fig. 9d). Some data points were observed falling on the dolomite and quartz lines. The data points falling on the dolomite line is due to the presence of the dolomitization within the Yamama Formation. However, the data points falling on the quartz line likely due to the abundance of peloids in the most recognized facies, where peloids acted similarly to quartz. Another reason for that is the gas affect which can cause a shift in the density-neutron cross-plot^26^. Additionally, some anomalous data in well logs can also be present. The major porosity values fall between 5 and 20%, with some data points that show porosity more than 20%.

#### Lithocodium-Bacinella facies (MF2 and MF4)

Lithocodium aggregatum, as described by Elliott^40^ in 1956, and Bacinella irregularis, identified by Radoičić^41^ in 1959, are mysterious microorganisms known for their encrusting nature. These enigmatic life forms were once widely distributed across Mesozoic carbonate platforms but have since vanished from the natural world^42^. They are frequently encountered within sedimentary rocks dating back to the Middle Jurassic through the Early Cretaceous era, spanning a timeframe of approximately 174 to 100 Ma. Lithocodium-Bacinella specimens are commonly associated with coral, sponge, and microbial reef frameworks. Additionally, they can contribute to the formation of oncoids in lagoonal and shoal environments. Recognizing the pivotal role of this facies in regulating the reservoir quality of the Yamama Formation within this study, it is imperative to emphasize the significance of the algal Lithocodium-Bacinella facies.

Lithocodium aggregatum is a calcareous alga known for its encrusting growth habit. It typically forms intricate, branching structures composed of small, rounded or irregularly shaped calcareous segments closely packed together. These segments may resemble small, button-like structures, and the surface of Lithocodium aggregatum often exhibits a granular or textured appearance due to numerous calcified layers. It can be found growing on various substrates, including rocks, shells, and other organisms^40,42,43^. Bacinella irregularis, another calcareous organism, is characterized by its fan-shaped or fan-like structures. It comprises fan-shaped plates or blades radiating outward from a central point. These blades can be thin and delicate, featuring a finely textured surface. The fan-shaped structures vary in size, and they often overlap or intertwine, forming intricate patterns (Fig. 6). Like Lithocodium aggregatum, Bacinella irregularis is commonly associated with other marine organisms, providing valuable insights into ancient marine environments^41–43^.

Lithocodium-Bacinella has been identified in various locations worldwide, including, Barremian Hautervian Ratawi Formation in South Iraq^19^, Hauterivian formations in Southeast France^44^, Abu Dhabi Onshore Field in the Lekhwair, Kharaib^45^, and Lower Shuaiba Formations, Aptian deposits in Oman^42^, and Late, Valanginian–Hauterivian in the Fahliyan Formation, eastern Persian Gulf Basin^38^. Their geographic range further extends from the southern USA and Mexico to NW Africa, the Iberic Plate, and across the Tethyan realm to China^46–48^. The Lithocodium-Bacinella were also found in the Lower Aptian of the western Maestrat Basin (Spain)^49^. Lithocodium typically thrived in a paleoenvironment characterized by warmth, full marine conditions, ample oxygenation, abundant calcium carbonate, and a mid-shelf sea setting. These conditions point to a shallow, warm environment with normal marine salinity, making it well-suited for the growth of Lithocodium aggregatum^43^.

The reservoir units YB1, YB2 and YC which form as the predominant reservoir units (along with YB3) within the Yamama Formation, are predominantly composed of Lithocodium-Bacinella facies (MF2 and MF4). MF2 and MF4 facies represent 45% of the cored intervals (Fig. 9a). The dissolution has played important role in improving the reservoir quality in this facies types of facies (Figs. 4c–f, 5e and 7d, and 6). The high porosity of Lithocodium-Bacinella algal facies is a result of their biogenic origin, encrusting growth habit, sediment-trapping capabilities, diagenesis, and their presence in ancient reef and lagoonal environments. These characteristics make them significant reservoir rocks and provide valuable insights into past marine ecosystems^50,51^. Since Lithocodium-Bacinella grows in lagoonal and reefal environments, the dissolution due to reactions between sediments and marine water is a crucial factor in the formation and modification of porosity in these types of facies. This process can result in the creation of both primary and secondary porosity and contributes to the overall heterogeneity of porosity within these rocks. The early Mg-calcite cement (isopachous) formed rims around grains and likely protected the porosity from compaction (Fig. 5c)^1,51^.

The vertical and lateral dimensions of Lithocodium-Bacinella lithosomes exhibit considerable variation, ranging from meter-scale build-ups to massive beds up to 8 m in thickness, and geo-bodies as high as 40 m^42,52,53^. Lithocodium-Bacinella can be characterized by their massive appearance, distinctly differing from adjacent bedded lagoonal facies^54^. In this study MF2 and MF4 formed thickness of 39 m and 37 m in YB2 and YC, respectively. Evidence from the seismic section shows that there are build ups within the Yamama Formation, where the seismic reflectors are distorted and not continuous in most of the formation’s parts (Fig. 2a). Similar carbonates build ups were found by Hillgartner et al.^15^. These build ups are highly likely to be due to the Lithocodium-Bacinella sediments. Therefore, targeting these build ups, especially, to the northeast part of Ah’Dimah-1 well is beneficial for developing the Yamama Formation in Ah’Dimah Oilfield.

#### A comparison with previous studies

Mohsin et al.^55^ briefly mentioned the existence of Lithocodium-Bacinella within the Yamama Formation, without providing substantial evidence or emphasizing the significance of these organisms in shaping the formation’s reservoir quality. Khazaal and Shakir^56^, in their biostratigraphy study, conducted in Luhais Oilfield, did acknowledge the presence of Lithocodium-Bacinella. However, Jameel and Al-Zaidy^57^ were unable to identify the Lithocodium-Bacinella facies during their investigation of the Yamama Formation in Ah’Dimah Oilfield. Their findings primarily indicated the existence of oolitic grainstone facies without clear substantiation, as these facies appeared more consistent with peloids and cortoids rather than oolitic in nature.

Our study confidently asserts the presence and highlights the paramount importance of the Lithocodium-Bacinella facies in influencing the reservoir quality of the Yamama Formation. This facies formed the best reservoir quality in Yamama Formation. Similarly, this facies was also noted as the best reservoir unit in the upper Fahliyan Formation, in the Persian Gulf^38^. This study marks the inaugural instance where the Lithocodium-Bacinella is proposed as one of the main identifiable facies within the Yamama Formation, located in the southern region of Iraq.

Noteworthy^6,8,58^ have stated the occurrence of oolitic limestone within the Yamama Formation, in the Ratawi Oilfield. Similarly, Idan et al.^24^ have reported the presence of oolitic limestone in the Yamama Formation, in the Majnoon Oilfield. In a separate study, Chafeet^59^ presented the results those discussed by Sadooni^23^, asserting the existence of oolitic limestone in the Yamama Formation, specifically within the West Qurna Oilfield. However, no clear evidence supporting the presence of oolitic limestone was provided in Chafeet’s study. Similarly, Handhal et al.^60^ did not show definitive proof of the occurrence of oolitic limestone within the Yamama Formation. In contrast, this study claims the absence of oolitic limestone in the Yamama Formation in the studied field. It is noteworthy that none of the aforementioned studies mentioned the Lithocoduim-Bacinella algae facies in their studies.

## Conclusions

A comprehensive analysis was conducted, combining well logs and petrographic analysis to evaluate the microfacies roles controlling the heterogeneity of the Yamama reservoir in south Iraq. The findings of this study have led to several key conclusions:

The formation is heterogenous and is divided into four reservoir (YA-YD) that separated by four non-reservoir units (BA-BD). A total of seven distinct microfacies were identified. These are bioclastic wackestone (MF1), Lithocodium-Bacinella float/boundstone (MF2), peloidal cortoid intraclast grainstone (MF3), reefal bioclastic rudstone (MF4), bioclastic foraminiferal wacke/packstone (MF5), miliolidal pack/grainstone (MF7), and spiculitic foraminiferal wackestone (MF7). According to their reservoir properties, Lithocodium-Bacinella is dominant the MF2 and MF4 facies and form 45% from the studied intervals. MF2 and MF4 formed good reservoir units, along with the grain supported MF3 facies. While the mud supported facies like MF1, MF5, and MF7 as well as MF6 formed the non-reservoir units.

The formation deposited in a shallow water carbonate ramp ranging from the lagoon to outer ramp. A Clear coincidence between the reservoir units and the depositional environment. Where, the reservoir units deposited in the shoal area (Backshoal, shoal center and shoalslope). The non-reservoir units deposited in the lagoon, middle and outer ramp. Therefore, the reservoir influenced by sea level fluctuations. The Lithocodium-Bacinella float/boundstone and reefal bioclastic rudstone form build-ups within reefal patches and of significant interest for the development of the Yamama Formation. Special attention should be directed towards areas northeast of the Ah’Dimah Oilfield for further exploration and development efforts.

The diagenetic processes significantly affected the formation. The early dissolution created vugs and channels, thereby enhancing the porosity. However, the presence of cements including syntaxial and calcite blocky cement occluded the pore spaces and deteriorated porosity. Early isopachous cement helped protect sediments from compaction, which had a greater influence on the fine-grained facies compared to the coarse-grained facies.

## Electronic supplementary material

Below is the link to the electronic supplementary material.

Supplementary Material 1

## Data Availability

The datasets generated and/or analyzed during this study are not publicly available because the data are for petroleum company, not normal data. the final figures are allowed for public. Therefore, the data that support the findings of this study are available from the corresponding author, but restrictions apply to the availability of these data, which were used under license for the current study, and so are not publicly available. The final version of data is however available from the corresponding author. in “Data Availability” section of this manuscript.
