# Author Correction: Microfacies impacts on reservoir heterogeneity of early Cretaceous Yamama carbonate reservoir in South Iraq

**DOI:** 10.1038/s41598-025-88030-3

**Published:** 2025-02-12

**Authors:** Abbas Mohammed, Felicitász Velledits

**Affiliations:** 1 Institute of Exploration Geosciences, University of Miskolc, Miskolc, 3515 Hungary; 2 Fields Division, Geology Department, Missan Oil Company, Misan, 62001 Iraq

Correction to: *Scientific Reports* 10.1038/s41598-024-74640-w, published online 15 October 2024

The original version of this Article contained an error in the Tectonic and geologic setting section.

“This cycle includes three maximum flooding surfaces. These are J110, K30, K20 and K10^12^.”

now reads:

“This cycle includes three maximum flooding surfaces. These are K30, K20 and K10^12^.”

In addition, in the legend of Figure 5,

“All photos are under crossed polarized light (XPL), except **b** and **a** (under plane polarized light PPL).”

now reads:

“All photos are under crossed polarized light (XPL), except **b** and **e** (under plane polarized light PPL).”

The original Article has been corrected.

