# Supplementary material for: Microfacies impacts on reservoir heterogeneity of early Cretaceous Yamama carbonate reservoir in South Iraq
Source: Sci Rep. 2024 Oct 15;14:24184. doi: 10.1038/s41598-024-74640-w (PMC11480476; doi:10.1038/s41598-024-74640-w)
Supplement: Supplementary file 1 — Supplementary Material 1 [file 41598_2024_74640_MOESM1_ESM.pdf]

Microfacies impacts on reservoir heterogeneity of early Cretaceous Yamama carbonate reservoir in South Iraq

Abbas Mohammed[1,2,*] and Felicitász Velledits[1]

[1]Institute of Exploration Geosciences, University of Miskolc, 3515, Miskolc, Hungary

[2]Geology Department, Fields Division, Misan Oil Company, 62001, Misan, Iraq

[*]Corresponding: mohammed.abbas@uni-miskolc.hu & goldengeo.87@gmail.com

[*]ORCID: https://orcid.org/0000-0003-4813-369X

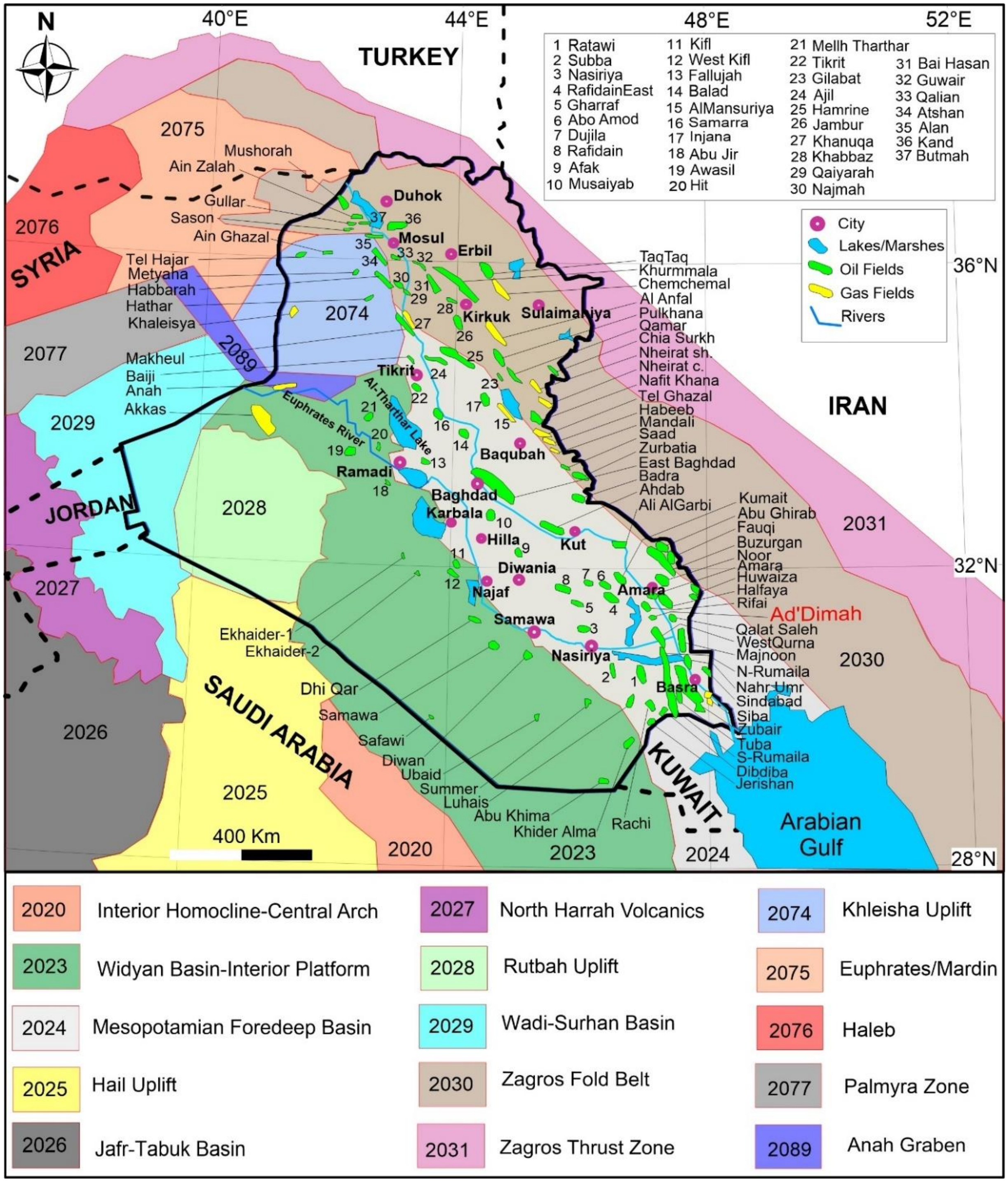

Supplementary Figure 1S. Iraqi tectonic map, including most of the oil/gas fields of Iraq (compiled from[9,16,17] and created using Surfer16.3.408 https://www.goldensoftware.com/). The studied field is highlighted with red color.

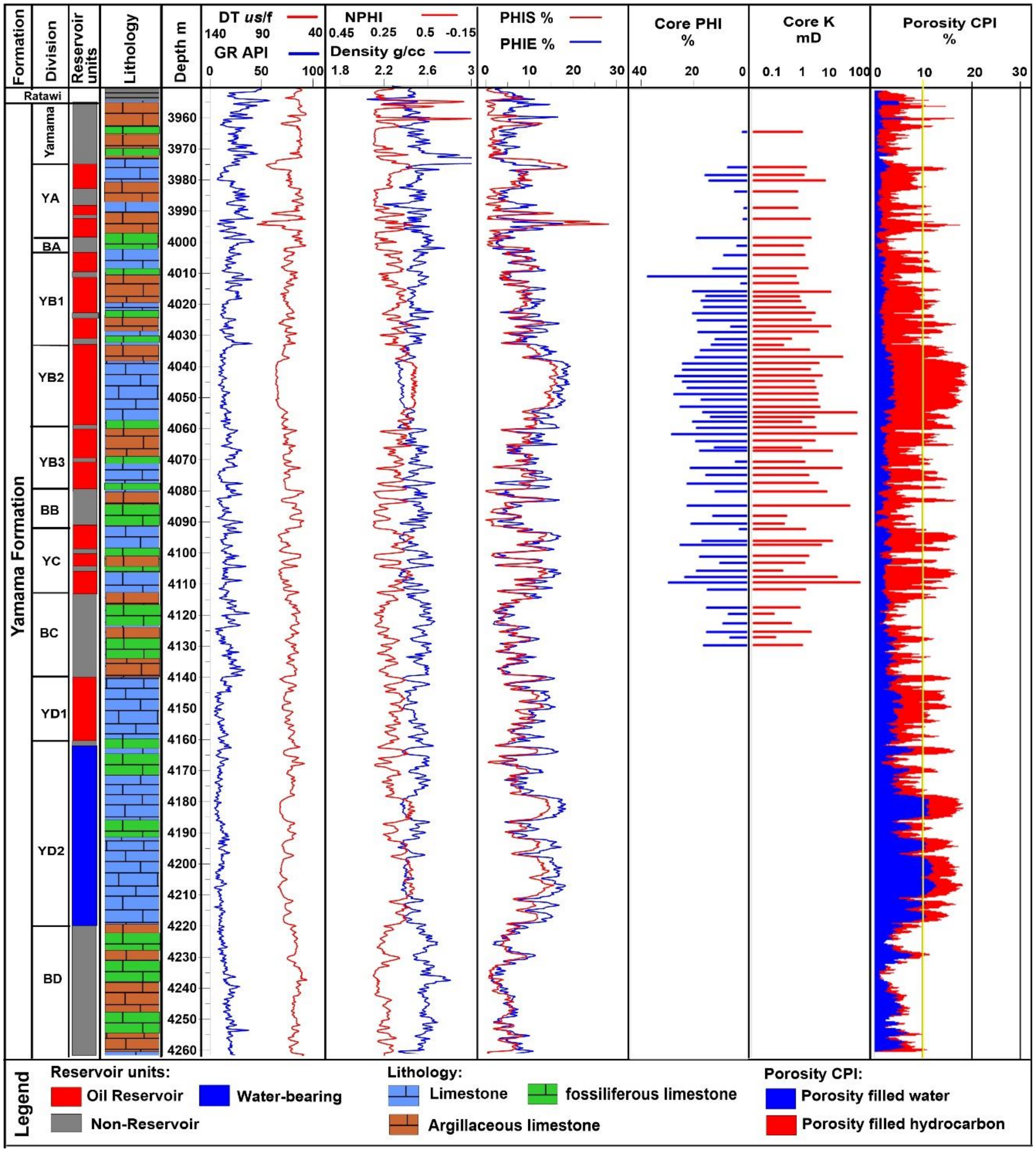

Supplementary Figure 2S. Well logs interpretation of the Yamama Formation in Ah'Dimah Oilfield shows gamma-ray, sonic, density, neutron, effective porosity (PHIE), sonic-derived porosity (PHIS), core porosity (PHI), permeability (K) measurements, and the porosity CPI obtained from the computed process interpretation (CPI), the porosity cutoff is 10%.
